# AbobotulinumtoxinA (Dysport^®^) Improves Function According to Goal Attainment in Children With Dynamic Equinus Due to Cerebral Palsy

**DOI:** 10.1177/0883073816686910

**Published:** 2017-01-09

**Authors:** Ann Tilton, Barry Russman, Resa Aydin, Umit Dincer, Raul G. Escobar, Sehim Kutlay, Zbigniew Lipczyk, Juan Carlos Velez, Anne-Sophie Grandoulier, Anissa Tse, Philippe Picaut, Mauricio R. Delgado

**Affiliations:** 1 LSUHSC and Children’s Hospital New Orleans, New Orleans, LA, USA; 2 Shriner’s Hospital for Children, Portland, OR, USA; 3 Istanbul University, Istanbul, Turkey; 4 GATA Haydarpasa Training Hospital, Istanbul, Turkey; 5 Unit of Neurology, Division of Pediatrics, Medical School, Pontificia Universidad Catolica de Chile, Santiago, Chile; 6 Department of PMR, Ankara University, Faculty of Medicine, Ankara, Turkey; 7 B and L–Specialist Medical Centre, Lodz, Poland; 8 Club De Leones Cruz Del Sur Rehabilitation Center, Punta Arenas, Chile; 9 Ipsen, Les Ulis, France; 10 University of Texas Southwestern Medical Center and Texas Scottish Rite Hospital for Children, Dallas, TX, USA

**Keywords:** abobotulinumtoxinA, Dysport, cerebral palsy, equinus foot, goal attainment scaling

## Abstract

This secondary analysis of a large (n = 241), randomized, double-blind study evaluated the efficacy of 2 doses of abobotulinumtoxinA + standard of care (SOC) versus placebo + SOC in enabling children with dynamic equinus due to cerebral palsy to achieve their functional goals using Goal Attainment Scaling. Most parents/caregivers selected goals targeting aspects of gait improvement as most relevant. Mean (95% confidence interval) Goal Attainment Scaling T scores at week 4 were higher for both abobotulinumtoxinA groups versus placebo (treatment difference vs placebo: 10 U/kg/leg: 5.32 [2.31, 8.32], *P* = .0006, and 15 U/kg/leg 4.65 [1.59, 7.71], *P* = .0031). Superiority of both abobotulinumtoxinA doses versus placebo was maintained at week 12. Best goal attainment T scores were higher in the abobotulinumtoxinA groups versus placebo for the common goals of improved walking pattern, decreased falling, decreased tripping, and improved endurance. These findings demonstrate that single injections of abobotulinumtoxinA (10 and 15 U/kg/leg) significantly improved the ability of pediatric cerebral palsy patients to achieve their functional goals.

Spasticity is a prevalent and frequently disabling motor disorder in children with cerebral palsy. Spasticity of the gastro-soleus muscle complex is common and often results in equinus foot posturing during the stance phase of gait.^1^ This causes patients to frequently trip, fall, and also interferes with shoe wear and use of orthosis. Treatment of spasticity in these muscles aims to improve gait patterns, thereby improving motor function and mobility. When used in conjunction with standard treatment such as physical therapy and lower extremity orthotics, botulinum toxin type A injections may prevent the development of fixed contractures, correct abnormal gait patterns, and even delay and/or reduce the need for surgical interventions.^2^ Clinical guidelines now recommend that botulinum toxin type A should be offered as an effective and generally well-tolerated treatment for localized/segmental spasticity in children and adolescents with cerebral palsy.^3–6^

Although previous studies provide evidence on the safety, tolerability and efficacy of botulinum toxin type A injections, many of these studies used reduction in spasticity and muscle tone as their main outcome measure.^7^ It has been widely suggested that efficacy assessments should also take into account the impact of the intervention on patient function and meaningful goals in the context of the patient’s own life.^8^ In the context of rehabilitation, one way to assess the functional impact of a treatment is to assess how well the treatment enables attainment of functional goals that are important to the patients and families.^9^ Goal setting is held to be a central component of effective multidisciplinary rehabilitation practice^6,10^ where the treating physician or therapist engages the patient and/or their parents/guardians in a discussion of realistic goals and prioritizes the aims of treatment. In clinical practice, the goals of botulinum toxin type A therapy can vary widely for each individual patient. Goal attainment scaling is a method of integrating attainment in a number of individually set goals into a single goal attainment score, such that the effectiveness of treatment can be easily compared, regardless of the goals chosen.^11^

We have previously reported the primary efficacy and safety results of this large international phase III study, which showed that treatment with abobotulinumtoxinA (Dysport^®^; Ipsen Biopharmaceuticals, Wrexham, UK) reduced muscle tone and spasticity compared to placebo in children with spasticity associated with cerebral palsy.^12^ In this report, we focus on the impact of abobotulinumtoxinA treatment on the ability of children with dynamic foot equinus to achieve their functional goals.

## Methods

### Study design

This was a double-blind, prospective, randomized, placebo-controlled, single-dose study (NCT01249417), full details of which have been previously published.^12^ Institutional review boards at the participating sites approved the protocol, and the trial was executed in accordance with the Declaration of Helsinki and International Conference on Harmonization Good Clinical Practice Guidelines.

In brief, this multicenter study included children (aged 2-17 years) with equinus foot positioning during stance phase of gait due to spastic cerebral palsy.^13^ Patients were required to be ambulatory (GMFCS Level I-III) and had to have a derived Modified Ashworth Scale score ≥2 as well as a Tardieu Scale spasticity grade (Y) of 2 to 4 (2 = catch and release, 3 = fatigable clonus, 4 = nonfatigable clonus) and a spasticity angle [X] of ≥10° at the ankle joint of the most affected limb to be injected. Patients could be botulinum toxin–naïve or previously treated, but the last botulinum toxin injection for any condition must have been >6 months prior to study entry. Key exclusion criteria were a nonambulatory status, a fixed-ankle flexor myocontracture (defined by a passive range of motion angle by the Tardieu Scale [XV1] of ≤80° in ankle dorsiflexion), severe athetoid/dystonic movements in the targeted leg(s), a significant leg length difference (>2 cm), treatment with any medication that interferes with neuromuscular function ≤30 days prior to study treatment. Patients were also excluded if they had previous surgery for lower limb spasticity, previous injections with alcohol and/or phenol, or serial lower-extremity casting within the past 12 weeks.

Eligible patients were assessed at baseline and randomized in a ratio of 1:1:1 to a single injection of abobotulinumtoxinA 10 U/kg/leg, abobotulinumtoxinA 15 U/kg/leg, or placebo and were stratified according to age (2-9 years and 10-17 years) and botulinum toxin–naïve or nonnaïve status. The abobotulinumtoxinA doses and schedule used have been previously established as effective.^14^ All patients received their current standard of care (SOC); established physiotherapy and/or orthotic regimes were permitted provided they had begun >1 month prior to study start and were maintained at the same level throughout the study.

Following treatment administration, patients attended follow-up visits at weeks 4 and 12. Additional visits were permitted at week 16, for patients who in the clinical judgment of the investigator did not require retreatment at week 12; at week 22, for patients who did not require retreatment at week 16; and at week 28, for patients who did not require retreatment at week 22.

### The Pediatric Goal Attainment Scale for Dynamic Foot Equinus

In this study, we used a list of goals that has been specifically developed for use in pediatric studies of botulinum toxin type A for dynamic foot equinus (B.R., unpublished data, 2009). Investigators and parents/guardians (and where age-appropriate the patient themselves) agreed on a total of 1 to 3 goals prior to study treatment, per published Goal Attainment Scaling methodology.^10^ There was an emphasis on setting SMART (specific, measurable, achievable, realistic, and timed) goals, and all injectors were trained in applying Goal Attainment Scaling methodology and the setting of SMART goals through a series of workshops. If more than one goal was chosen, the parents/guardians rated one as “very important” and ranked the others accordingly. Likewise, investigators rated the level of difficulty they expected for achieving the set goal. The tool included 12 predefined goals covering active and passive function as well as pain (Table 1).

**Table 1.** Frequency of Goal Choice at Baseline in Rank Order of Parent Preference.

Goals chosen at baseline^a^	Placebo group (n = 77), n (%)	AbobotulinumtoxinA 10 U/kg/leg group (n = 79), n (%)	AbobotulinumtoxinA 15 U/kg/leg group (n = 79), n (%)	All patients (n = 235), n (%)
Improved walking pattern	54 (70)	48 (61)	63 (80)	165 (70)
Improved balance	19 (25)	31 (40)	26 (33)	76 (32)
Decreased frequency of falling	25 (33)	22 (28)	26 (33)	73 (31)
Decreased frequency of tripping	13 (17)	16 (20)	17 (22)	46 (20)
Improved endurance	11 (14)	18 (23)	11 (14)	40 (17)
Decreased foot pain	10 (13)	6 (8)	5 (6)	21 (9)
Improved walking speed	3 (4)	6 (8)	9 (11)	18 (8)
Improved tolerance of ankle foot orthosis	5 (7)	7 (9)	4 (5)	16 (7)
Improved cosmesis (looks better)	7 (9)	2 (3)	5 (6)	14 (6)
Increased duration of shoe wear	1 (1)	1 (1)	2 (3)	4 (2)
Improved ease of putting on ankle foot orthosis	2 (3)	0 (0)	1 (1)	3 (1)
Other^b^	18 (23)	10 (13)	12 (15)	40 (17)

^a^Parents (and/or patients) could choose between 1 and 3 goals at baseline.

^b^If “other” was chosen, the goal had to be specified.

Goal attainment for the chosen goals was assessed by the parents/caregivers at week 4 and at weeks 12, 16, 22, and 28. Based on parental input, investigators rated achievement on a predefined 5-point scale (–2 = much less than expected, –1 = somewhat less than expected, 0 = expected outcome, +1 = somewhat more than expected, and +2 = much more than expected). Both parents/children and investigators were blinded to study treatment allocation.

### Statistical Analysis

Analyses were performed on the intention-to-treat population, including all randomized participants who received ≥1 injection of study treatment and had recorded Modified Ashworth Scale scores at baseline and week 4.

Raw Goal Attainment Scaling scores were transformed into a standardized measure (T-score) with a mean of 50 and an SD of 10.^15^ In this system, a Goal Attainment Scaling T score of 50 represents goals achieved as expected. Scores below 50 reflect underattainment of goals and scores greater than 50 represent overattainment of goals. Mean Goal Attainment Scaling T scores at week 4 were analyzed as a key secondary efficacy measure using an analysis of variance model with randomization stratification factors (age range and botulinum toxin treatment status at baseline) and treatment center included as fixed effects. Mean Goal Attainment Scaling T scores at week 12 were analyzed in the same way as a tertiary outcome. Goal Attainment Scaling T scores at later weeks were not analyzed because of underpowering.

Other exploratory outcomes included T scores for each individual goal and “responder” analyses (where response is defined as achieving a Goal Attainment Scaling T score >50) assessed at weeks 4, 12, 16, and 22. In addition, to account for the fact that some goals take longer to achieve than others, we performed an analysis of “best” goal attainment (Goal Attainment Scaling T score and individual goals). This analysis focused on the goal ranked as “most important” by the parents/guardians and used the highest goal attainment score, regardless of when this best score was achieved.

## Results

### Patient Disposition

The study began on July 5, 2011, and was completed on June 25, 2014. The intention-to-treat population included 235 (placebo, n = 77; abobotulinumtoxinA 10 U/kg/leg, n = 79; abobotulinumtoxinA 15 U/kg/leg, n = 79) of the 241 randomized patients. The majority of abobotulinumtoxinA patients met criteria for retreatment at the week 16 and 22 visits (33.5% and 22.8%, respectively), whereas 17.7% of all abobotulinumtoxinA-treated patients met retreatment criteria at week 28 or later.

Baseline characteristics have previously been presented.^12^ In brief, 141 patients were male (60%); the majority of patients were aged 2 to 9 years (85%), and 15% were aged 10 to 17 years. Overall, 56% of patients were GMFCS level I, 33% were GMFCS level II, and 11% were GMFCS level III; 50% had hemiparesis, 44% had diparesis, and 6% had tetraparesis. The majority (88.5%) of patients used concomitant orthosis and/or physiotherapy, prior to and during the study.

### Baseline Goal Choice

At baseline, the 235 patients in the intention-to-treat population set a total of 516 goals (mean 2.2 goals per patient). The most frequently chosen goals were improved walking pattern (70% of patients), improved balance (32%), decreased frequency of falling (31%), decreased frequency of tripping (20%), and improved endurance (17%) (Table 1). When improved walking pattern was chosen as a treatment goal, the majority of parents rated this goal as “very important” (69% in the placebo group, 67% in the abobotulinumtoxinA 10 U/kg/leg group, and 75% in the abobotulinumtoxinA 15 U/kg/leg group).

### Goal Attainment Scaling T Scores at Weeks 4 and 12

**Figure 1.**
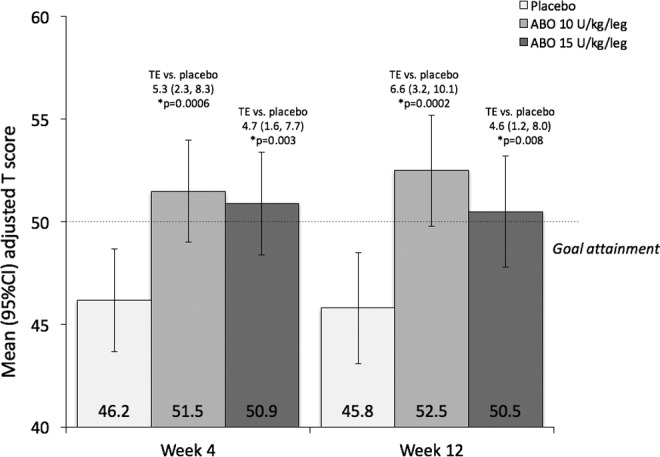
GAS T scores at weeks 4 and 12. Columns represent adjusted mean (95% confidence interval) GAS T scores. Adjusted LS Means were obtained from an analysis of covariance on the change from baseline with treatment, baseline score, age range at baseline, botulinum toxin status at baseline, and center as covariates. ABO, abobotulinumtoxinA; GAS, Goal Attainment Scaling; TE, adjusted LS mean (95% confidence interval) treatment effect versus placebo.

Whereas patients in the abobotulinumtoxinA groups showed expected goal attainment (ie, Goal Attainment Scaling T scores >50) at weeks 4 and 12, patients in the placebo group did not reach the expected level. The adjusted mean treatment differences for active treatment (both doses) versus placebo were also significant at both time points (Figure 1).

Analysis of best goal attainment also favored active treatment. Patients in both the abobotulinumtoxinA 10-U/kg/leg and 15-U/kg/leg groups achieved higher mean best goal attainment scores compared with the placebo group (best Goal Attainment Scaling T scores of 55.5, 54.8, and 48.8, respectively).

### Analysis of Individual Goals

Taken overall, patients in the active treatment groups were more likely to attain their functional goals (ie, score ≥50) than patients in the placebo group. Of the 5 most commonly selected goals, best goal attainment T scores were higher in the active treatment groups versus placebo, with the exception of “improved balance,” for which there was a similar mean T score across the 3 groups (Table 2). Analysis of the other less frequently chosen goals was less informative because of the small sample size (<10% of patients).

**Table 2.** Responder Analyses for Achievement of Primary Goal and for the Five Most Commonly Chosen Individual Goals.^a^

	Placebo group (n = 77)	AbobotulinumtoxinA 10 U/kg group (n = 79)	AbobotulinumtoxinA 15 U/kg group (n = 79)
Primary goal achievement (at any time during study), n (%)	47/76 (62)	62/79 (79)	60/79 (76)
Individual goal analysis			
Improved walking pattern			
Best goal attainment T score, mean (SD)	45.4 (8.8)	54.2 (9.6)	52.7 (10.0)
Responder rate at week 4, n (%)	21/53 (40)	38/48 (79)	38/63 (60)
Responder rate at week 12, n (%)	19/49 (39)	31/43 (72)	38/60 (63)
Improved balance			
Best goal attainment T score, mean (SD)	47.9 (7.9)	51.3 (9.2)	48.5 (10.8)
Responder rate at week 4, n (%)	10/19 (53)	18/29 (62)	10/26 (39)
Responder rate at week 12, n (%)	9/16 (56)	16/26 (62)	14/25 (56)
Decreased frequency of falling			
Best goal attainment T score, mean (SD)	50.4 (10.6)	59.1 (11.5)	56.5 (10.6)
Responder rate at week 4, n (%)	14/25 (56)	18/22 (82)	18/26 (69)
Responder rate at week 12, n (%)	8/19 (42)	18/20 (90)	17/24 (71)
Decreased frequency of tripping			
Best goal attainment T score, mean (SD)	51.5 (12.8)	52.5 (10.6)	57.1 (9.9)
Responder rate at week 4, n (%)	6/13 (46)	9/16 (56)	13/17 (77)
Responder rate at week 12, n (%)	8/13 (62)	9/14 (64)	14/16 (88)
Improved endurance			
Best goal attainment T score, mean (SD)	50.0 (8.9)	56.1 (7.8)	58.2 (10.8)
Responder rate at week 4, n (%)	6/11 (55)	13/18 (72)	7/11 (64)
Responder rate at week 12, n (%)	5/11 (46)	14/16 (88)	10/11 (91)

Abbreviation: SD, standard deviation.

^a^Best goal attainment total score for each patient was assessed using the best score attained for each goal at any time during the study. Patients who completed the study or withdrew are counted as missing at subsequent visits.

### Responder Analyses

The proportion of patients whose primary goal reached expected or better than expected outcome during the study (best goal attainment) was significantly higher in the abobotulinumtoxinA 10-U/kg/leg group versus placebo (79% vs 62%; *P* = .03). Patients in the abobotulinumtoxinA 10 U/kg/leg group were more than twice as likely to reach their goal as the placebo group (odds ratio [95% confidence interval] of 2.2 [1.1, 4.6]). Although the proportion of responders in the abobotulinumtoxinA 15 U/kg/leg group was also numerically higher (76%) than the placebo group, the difference was not statistically significant (*P* = .06). Responder analyses showed that results for 4 of the 5 most commonly chosen individual goals were in line with the individual T score analyses, with a generally higher response rate in the active treatment groups versus placebo. However, the response rate for the goal of “improved balance” was smaller in the abobotulinumtoxinA 15 U/kg/leg group versus the other groups (Table 2).

## Discussion

We have previously reported that single injections of abobotulinumtoxinA significantly improve muscle hypertonia and spasticity, resulting in a better overall global clinical outcome.^12^ The results of these secondary and exploratory analyses of goal attainment support the overall findings and confirm that treatment with abobotulinumtoxinA improved the ability of patients to achieve their treatment goals versus placebo.

Although the use of Goal Attainment Scaling as an outcome measure is now well established in studies of adult spasticity^10,15^ and is validated for use in all age groups,^16^ it is a relatively underexplored concept in pediatric trials.^17^ To our knowledge, this is one of the largest pediatric cerebral palsy studies to include Goal Attainment Scaling as an outcome measure. The implementation of Goal Attainment Scaling in our protocol involved extensive training to ensure that all investigators set goals in a consistent way. This process required us to develop our goal-setting and negotiation skills into a very structured process, which takes into account what is important to the patient and what the physician and therapist believe is achievable. In our experience, the use of Goal Attainment Scaling necessitated that we modify what could be very broad goals into predefined measurable steps that enabled a better judgment of treatment response. For example, instead of setting a goal of “improved walking pattern” in a child who is constantly toe walking, the use of Goal Attainment Scaling meant that this broad goal could be broken down into objective steps such as toe walking for 50% to 75% of the time (expected attainment), toe walking for 25% to 49% of the time (somewhat better than expected attainment), and not toe walking (much better than expected attainment). Although this approach admittedly involved more time at the initial visit, the early efforts invariably saved time during the follow-up visits.

Importantly, these data confirm our clinical experience of good functional benefits with botulinum toxin type A treatment but contradict an evidence base that has often failed to show significant efficacy of a botulinum toxin type A product in improving patient function.^17,18^ This may, in part, be because few previous study protocols have included the extensive training and standardization as described above.^19^ It may also reflect the fact that the use of Goal Attainment Scaling allows a range of assessment measures that are individualized to the patient (rather than one measure for all). The diagnosis of cerebral palsy is very diverse in terms of the impairments and limitations a child may experience (even within the limits of study inclusion criteria) and these differences emphasize the need for a more flexible and sensitive measure that evaluates the impact of treatment for children with cerebral palsy in a more meaningful fashion.^18^

In this study, we used a list of 12 predefined Goal Attainment Scaling goals that have previously been reported to be responsive to treatment and well accepted by patients and their families (B.R., unpublished data, 2009). Although the scale included a list of 12 predefined goals, it is clear that most parents/patients chose an improved walking pattern, improved balance, and/or decreased frequency of falling as very important or important goals. Because these types of goals typically require significant physical input (eg, strength and/or balance), our findings suggest that the injections “enabled” patients to progress toward reaching their goals (vs a direct effect of the toxin). Although other goals such as reduced foot pain and easier AFO use are often cited as reasonable “indications” for starting therapy with botulinum toxin type A,^5,6^ they were much less commonly chosen by the parents/patients themselves. Of note, in the 15 U/kg/leg group, the goal of “improved balance” was not attained at week 4. However, by week 12, the percentage of responders in the 15 U/kg/leg group had increased from 39% to 56%. The lack of improvement at week 4 was not unexpected (indeed, the results for the 10 U/kg/leg are the more surprising) since balance improvement usually requires improvements in strength and muscle coordination, which require additional physical input and take longer to achieve.

To account for the fact that some goals were expected to take more than 4 weeks after botulinum toxin therapy to achieve; we performed a preplanned analysis of the best scores for each goal during the study. In this second analysis, better goal attainment was achieved in both abobotulinumtoxinA groups as compared to placebo. The good results at week 12 clearly demonstrate that the duration of treatment benefit exceeds the time that the toxin is active in the muscle (maximum benefit from chemodenervation is usually achieved within 4-6 weeks). Indeed, it is notable that almost three-quarters (74%) of patients were retreated at 16 weeks or later, and almost 1 in 5 patients did not require retreatment until at least 28 weeks after abobotulinumtoxinA injection, suggesting that these patients were able to meet their goals of treatment over this extended period of time.

Strengths of the study include the large study size, multicenter design (which supports the benefits of this treatment approach across different cultures), the double-blind design that allowed assessment of Goal Attainment Scaling in a nonbiased way, and the significant training given to investigators to perform a standardized Goal Attainment Scaling assessment. Limitations include the lack of tailoring of the injection schema according to the patient profile and goals selected. This study only assessed the efficacy of a single injection cycle. Achievement of mobility goals require a long-term commitment to therapy,^5^ and further assessment of goal attainment in the long-term open-label extension study will provide better insights into the long-term efficacy of repeated injections.

In conclusion, this is the first placebo-controlled study to demonstrate that single injections of abobotulinumtoxinA (10 or 15 U/kg/leg) significantly improve the ability of pediatric patients with cerebral palsy to achieve their functional goals that are important to the patients and their families. Goal Attainment Scaling is a useful measure to include in the routine clinical assessment and management of children with cerebral palsy and has the potential to improve current practice. Realistic goals and expectations should be established with the patient and family before treatment, and the therapy should be tailored to their individual needs.
